# Evaluating the reliability and quality of retinal detachment educational content on TikTok and Bilibili: A cross-sectional content analysis

**DOI:** 10.1097/MD.0000000000050274

**Published:** 2026-08-14

**Authors:** Shengjin Tu, Kaiyin Tan, Jingtao Huang, Jinyang Peng

**Affiliations:** a Department of Ophthalmology, Central People’s Hospital of Zhanjiang, Zhanjiang, Guangdong, China; b Department of Sports Medicine and Rehabilitation, Peking University Shenzhen Hospital, Shenzhen, Guangdong, China.

**Keywords:** Bilibili, information quality, retinal detachment, short video, TikTok

## Abstract

Retinal detachment (RD) is a sight-threatening emergency where public awareness of early symptoms is critical. Bilibili and TikTok now serve as prominent health information channels, yet RD-related video content quality and reliability remain unassessed. This cross-sectional study systematically retrieved the top 150 videos using the keyword “retinal detachment” from Bilibili and TikTok in October 2025. Following exclusion criteria application, 224 videos underwent analysis. Two ophthalmologists independently assessed video quality and reliability using the Global Quality Scale (GQS), modified DISCERN, and *Journal of the American Medical Association* (JAMA) benchmark criteria. Analyses also covered content themes, uploader identity, and correlations with user engagement metrics. Among the 224 videos, overall quality was suboptimal (median scores: GQS = 2, modified DISCERN = 2, JAMA = 2). Treatment information dominated (67.86%), while content on symptoms (48.21%) and particularly prevention (32.59%) was scarce. Although overall scores were low, the distributions of GQS (*P* = .014) and JAMA (*P* = .002) scores differed significantly between self-identified professional and nonprofessional uploaders, despite identical group medians. A key finding was the negligible correlation between user engagement metrics and quality scores, indicating that popularity does not reflect content accuracy. Current RD information on short-form video platforms is characterized by generally low quality, significant content imbalance, and a disconnect between engagement and reliability. Urgent collaborative efforts among content creators, platform regulators, and healthcare institutions are needed to establish quality control standards, promote evidence-based content, and enhance public health literacy to leverage social media for improving RD outcomes.

## 1. Introduction

Retinal detachment (RD), a severe ocular emergency, typically presents with sudden visual field defects or an acute decline in visual acuity. Epidemiological data estimate an incidence of RD of roughly 1 to 2 cases per 10,000 persons annually, peaking in the sixth to seventh decades of life, with a markedly increased risk in individuals with high myopia.^[1–4]^ If not promptly treated, RD can rapidly induce photoreceptor apoptosis and irreversible vision loss, substantially impairing quality of life and imposing a heavy societal burden.^[5]^ Therefore, improving public awareness of early warning signs – such as a sudden increase in floaters, photopsia (flashes), or a curtain-like obscuration of the visual field – is crucial to promote timely medical consultation, improve prognosis, and reduce the overall disease burden.^[6,7]^

Digital media now serves as a primary source of health information for the public. Platforms like Bilibili and TikTok, with their vast user bases, algorithm-driven personalized recommendations, and highly interactive features, facilitate the rapid dissemination of medical knowledge.^[8–10]^ However, this convenience is accompanied by serious challenges regarding content quality and reliability. Prior studies in other disease areas, such as thyroid eye disease^[11]^ and knee osteoarthritis,^[12]^ have shown that while short-form videos achieve high engagement, the quality of information is often heterogeneous and carries a risk of misinformation. For time-sensitive conditions like RD that require urgent intervention, low-quality information can foster erroneous beliefs and mislead patients into inappropriate self-management, thereby delaying accurate diagnosis and definitive surgical treatment and potentially causing irreversible vision loss. To date, however, there is a dearth of systematic evaluations of short-video content related to RD.

This study aims to be the first to comprehensively assess the quality and reliability of RD short videos on the Bilibili and TikTok platforms using the Global Quality Scale (GQS), the modified DISCERN (mDISCERN) tool, and the *Journal of the American Medical Association* (JAMA) benchmark criteria. Employing a cross-sectional survey approach, we will delineate the strengths and shortcomings of the current health education content, thereby providing evidence-based recommendations for medical content creators, guidance to help the public identify trustworthy information, and empirical data to inform the optimization of early intervention strategies for RD and public health communication models.

## 2. Materials and methods

### 2.1. Video collection method

This cross-sectional study systematically gathered short videos on the Bilibili and TikTok platforms with the search keyword “retinal detachment” from October 2, 2025, to October 7, 2025. To reduce algorithmic influence from platform personalization, searches were performed without logging in. For both platforms, the initial sample comprised the top 150 videos from the default comprehensive ranking. Exclusion criteria were as follows: videos unrelated to RD; duplicate videos; and videos uploaded less than 1 week prior. Figure S1, Supplemental Digital Content 1, outlines the procedure in detail. We recorded detailed information on the final included videos (video title, uploader name, video length, likes, collections, shares, comments, and video content) and documented these in a Microsoft Excel spreadsheet.

### 2.2. Uploader characteristics

Based on prior literature, video uploaders were initially classified according to publicly available account information as self-identified professional uploaders or nonprofessional uploaders.^[11]^ For accounts presenting themselves as medical professionals, the original video and uploader pages were reviewed, and the claimed identities and specialties were cross-checked, where possible, using publicly accessible hospital or institutional webpages, professional profiles, and online curricula vitae. Platform profile descriptions and verification badges were treated as supporting information rather than definitive evidence of professional licensure. Because some accounts could not be independently linked to a named licensed professional, the term “self-identified professional uploaders” is used throughout this study. These uploaders were further subdivided into ophthalmologists, ophthalmologists practicing traditional Chinese medicine, and other specialists, including physicians in ultrasound and diagnostic imaging. Nonprofessional uploaders primarily comprised individual users, patients, and media professionals.

### 2.3. Video content evaluation

Video content was categorized by thematic area into epidemiology, etiology, symptoms, diagnosis, treatment, and prevention of RD.^[5]^ A single video may address more than one of these topics.

### 2.4. Quality and reliability assessment

Three validated tools were used in this study to assess the quality and reliability of short-form videos: GQS, the mDISCERN instrument, and JAMA benchmark criteria. The GQS (Table S1, Supplemental Digital Content 2) assesses overall video quality on a 5-point scale (1–5), with higher scores reflecting better overall quality.^[13]^ The mDISCERN instrument (Table S2, Supplemental Digital Content 3) is adapted from the original DISCERN tool developed for written health information and modified for video assessment. It assesses the reliability of content in 5 areas: impartiality, robustness, traceability, relevance, and clarity. Each of these areas receives a score between 0 and 1, for a total score between 0 and 5, where higher scores denote more reliability.^[14]^ The JAMA benchmark criteria (Table S3, Supplemental Digital Content 4) comprise 4 items – authorship, attribution, currency, and disclosure of conflicts of interest – used to appraise source credibility and informational accuracy; total scores vary between 0 and 4, with higher scores denoting more reliable information.^[15]^ All videos were independently rated by 2 ophthalmologists who had undergone standardized training. If the 2 raters’ scores differed by ≥ 1 point on any instrument, the discrepancy was considered significant, and a third senior ophthalmologist was invited to adjudicate; the adjudicator’s assessment was taken as the final score.^[12]^

### 2.5. Statistical analysis

Continuous variables were summarized using descriptive statistics. Data conforming to a normal distribution are presented as mean ± standard deviation, whereas non-normally distributed data are expressed as median and interquartile range. Categorical variables are reported as counts and percentages. Between-group comparisons were performed using the independent-samples *t* test for normally distributed variables and the Mann–Whitney *U* test for non-normally distributed variables. The Mann–Whitney *U* test compares the ranks of all observations and may detect between-group differences in the overall distributions even when the groups have the same median. Within the self-identified professional uploader group, GQS, mDISCERN, and JAMA scores were compared among ophthalmologists, ophthalmologists practicing traditional Chinese medicine, and other specialists. Because the scores were ordinal and subgroup sizes were markedly unequal, overall comparisons were performed using Monte Carlo permutation-based Kruskal–Wallis tests with 1,000,000 permutations. When an overall comparison was statistically significant, pairwise permutation-based Mann–Whitney *U* tests with Holm adjustment were performed. Epsilon-squared (ε^2^) was calculated as an effect-size estimate. The Spearman rank correlation coefficient (*r*) was used to assess associations between video quality scores (GQS, mDISCERN, and JAMA scores) and general metrics (video duration and numbers of likes, comments, shares, and saves/bookmarks). Correlation strength was classified as |*r*| < 0.2 (negligible), 0.2 to 0.4 (weak), 0.4 to 0.6 (moderate), 0.6 to 0.8 (strong), and >0.8 (very strong). Two-tailed *P* values < .05 were considered statistically significant. Data processing and the original analyses were performed using R version 4.3.3 (R Foundation for Statistical Computing, Vienna, Austria) and Zstats version 1.0 (Zstats Development Team, Hangzhou, China; available at www.zstats.net), while the additional permutation-based subgroup analyses were performed using Python 3.13.5 (Python Software Foundation, Beaverton).

### 2.6. Ethical considerations

This study exclusively analyzed publicly available videos from TikTok and Bilibili. It involved no interaction with platform users and collected no personally identifiable, clinical, or private information. Therefore, ethics committee or institutional review board approval was not required, and informed consent was not applicable.

## 3. Results

### 3.1. Video characteristics

The final analysis comprised 224 videos, whose attributes appear in Table 1. The median duration of these videos was 103.50 seconds (Q_1_ = 67.75, Q_3_ = 257.00), while the median counts of likes, collections, comments, and shares were 163.00 (Q_1_ = 45.75, Q_3_ = 721.25), 64.50 (Q_1_ = 19.75, Q_3_ = 260.50), 19.00 (Q_1_ = 4.00, Q_3_ = 103.50), and 24.00 (Q_1_ = 6.00, Q_3_ = 112.00), respectively. Regarding video quality and reliability, the median GQS score was 2.00 (Q_1_ = 2.00, Q_3_ = 3.00), and the median mDISCERN and JAMA scores were both 2.00 (Q_1_ = 2.00, Q_3_ = 2.00).

**Table 1:** The general information, content, quality, and reliability scores of the videos.

Variables	Total (n = 224)
General information	
Video length (s), M (Q_1_, Q_3_)	103.50 (67.75, 257.00)
Likes, M (Q_1_, Q_3_)	163.00 (45.75, 721.25)
Collections, M (Q_1_, Q_3_)	64.50 (19.75, 260.50)
Comments, M (Q_1_, Q_3_)	19.00 (4.00, 103.50)
Shares, M (Q_1_, Q_3_)	24.00 (6.00, 112.00)
Video content	
Epidemiology, n (%)	49 (21.88)
Etiology, n (%)	90 (40.18)
Symptoms, n (%)	108 (48.21)
Diagnosis, n (%)	72 (32.14)
Treatment, n (%)	152 (67.86)
Prevention, n (%)	73 (32.59)
Quality and reliability	
GQS score, M (Q_1_, Q_3_)	2.00 (2.00, 3.00)
mDISCERN score, M (Q_1_, Q_3_)	2.00 (2.00, 2.00)
JAMA score, M (Q_1_, Q_3_)	2.00 (2.00, 2.00)

GQS = Global Quality Scale, JAMA = *Journal of the American Medical Association* benchmark criteria, M = median, mDISCERN = modified DISCERN, Q_1_ = first quartile, Q_3_ = third quartile.

### 3.2. Comparison of features across platforms

The study encompassed 224 videos: 82 from Bilibili and 142 from TikTok. Table 2 compares the video characteristics between the 2 platforms. Video duration was significantly longer on Bilibili than on TikTok (*P* < .001). TikTok videos had significantly higher numbers of likes, collections, comments, and shares compared with Bilibili videos (*P* < .001), indicating stronger audience engagement on TikTok. In terms of video quality and reliability, the median GQS was 2.00 (Q_1_ = 2.00, Q_3_ = 3.00) for Bilibili and 2.00 (Q_1_ = 2.00, Q_3_ = 2.00) for TikTok; this difference was statistically significant (*P* < .001). The median mDISCERN and JAMA scores for both Bilibili and TikTok videos were 2.00 (Q_1_ = 2.00, Q_3_ = 2.00), with no significant interplatform differences (Fig. S2, Supplemental Digital Content 5).

**Table 2:** General information, quality, and reliability scores of RD videos on Bilibili and TikTok.

Variables	Bilibili (n = 82)	TikTok (n = 142)	*P* value
General information			
Video length (s), M (Q_1_, Q_3_)	174.50 (76.50, 510.00)	93.50 (62.25, 142.00)	<.001
Likes, M (Q_1_, Q_3_)	29.50 (4.00, 143.75)	259.00 (116.00, 845.75)	<.001
Collections, M (Q_1_, Q_3_)	24.00 (7.00, 114.00)	98.50 (37.00, 290.75)	<.001
Comments, M (Q_1_, Q_3_)	4.00 (0.00, 62.00)	29.50 (11.00, 128.00)	<.001
Shares, M (Q_1_, Q_3_)	9.00 (1.25, 48.75)	39.00 (14.00, 196.75)	<.001
Quality and reliability			
GQS score, M (Q_1_, Q_3_)	2.00 (2.00, 3.00)	2.00 (2.00, 2.00)	<.001
mDISCERN score, M (Q_1_, Q_3_)	2.00 (2.00, 2.00)	2.00 (2.00, 2.00)	.260
JAMA score, M (Q_1_, Q_3_)	2.00 (2.00, 2.00)	2.00 (2.00, 2.00)	.826

GQS = Global Quality Scale, JAMA = *Journal of the American Medical Association* benchmark criteria, M = median, mDISCERN = modified DISCERN, Q_1_ = first quartile, Q_3_ = third quartile, RD = retinal detachment.

### 3.3. Comparison of features across different video sources

**Figure 1.**
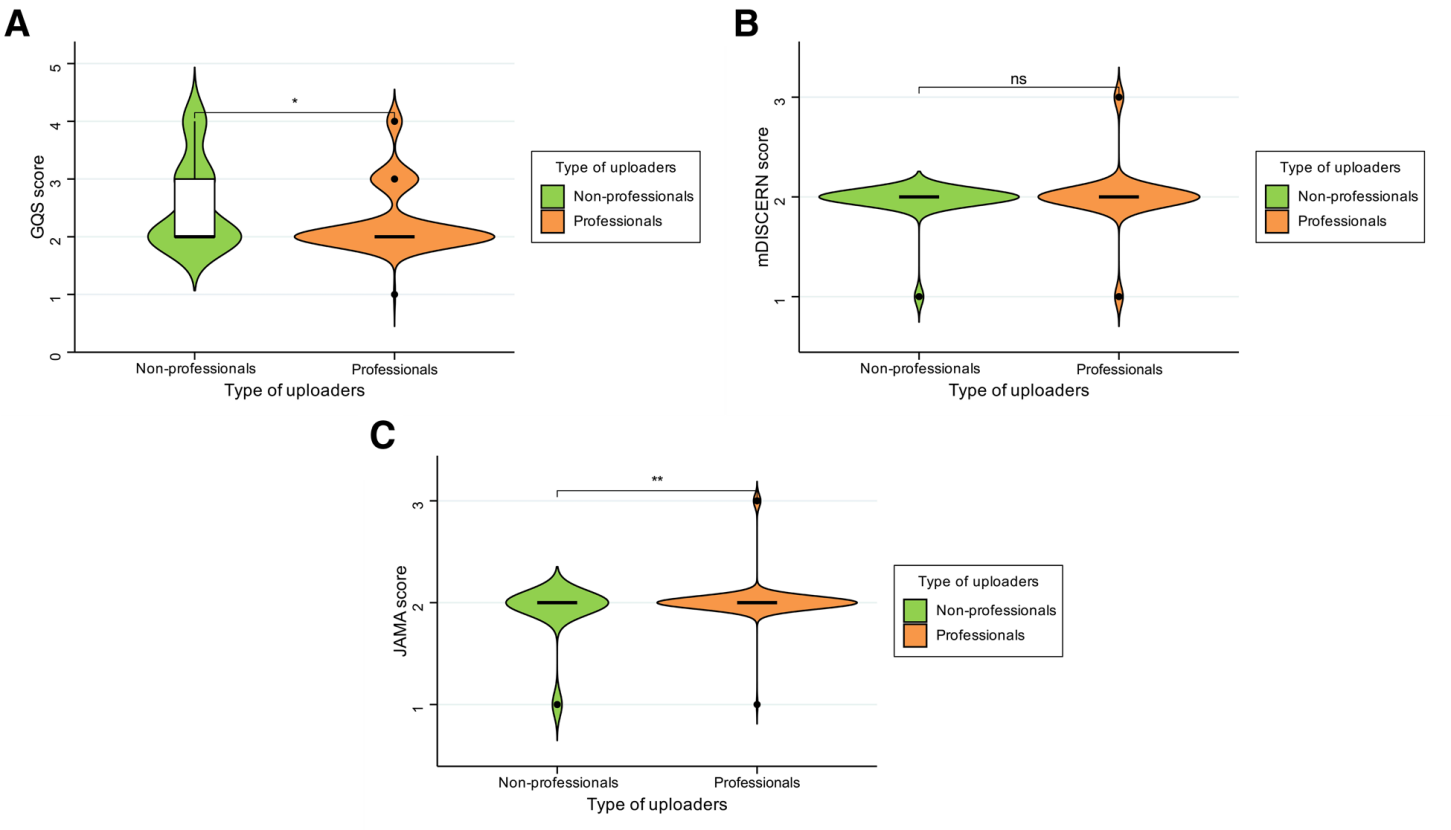
Comparison of quality and reliability scores between self-identified professional and nonprofessional uploader groups. (A) GQS score. (B) mDISCERN score. (C) JAMA score. GQS = Global Quality Scale, JAMA = *Journal of the American Medical Association* benchmark criteria, mDISCERN = modified DISCERN.

Of the 224 videos, 177 (79.02%) were uploaded by accounts categorized as self-identified professional uploaders, whereas 47 (20.98%) were uploaded by nonprofessional uploaders. On TikTok, self-identified professional uploaders contributed 96% of the videos and nonprofessional uploaders contributed 4%, whereas on Bilibili the 2 categories each contributed 50% (Fig. S3, Supplemental Digital Content 6). Table 3 shows that videos from self-identified professional uploaders were significantly shorter than those from nonprofessional uploaders and received more likes and collections. As detailed in Table 3 and illustrated in Figure 1, significant differences in video quality and reliability were observed between self-identified professional and nonprofessional uploaders. Although the median GQS score was 2.00 in both groups, the overall GQS score distributions differed significantly (*P* = .014). The median JAMA score was also 2.00 in both groups, but the overall JAMA score distributions differed significantly (*P* = .002). These results arose from differences in the frequencies and ranks of observations across score categories rather than from differences in the median values. The mDISCERN score distributions did not differ significantly between the groups (*P* = .317). Among the 177 videos uploaded by self-identified professional uploaders, 169 were uploaded by ophthalmologists, 3 by ophthalmologists practicing traditional Chinese medicine, and 5 by other specialists. The median GQS scores were 2.00 (Q_1_ = 2.00, Q_3_ = 2.00), 2.00 (Q_1_ = 2.00, Q_3_ = 2.00), and 3.00 (Q_1_ = 2.00, Q_3_ = 4.00), respectively. The overall GQS distributions differed among the 3 subgroups (*H* = 5.756, permutation *P* = .040, ε^2^ = 0.022). However, no pairwise comparison remained statistically significant after Holm adjustment (all adjusted *P* > .05). The median mDISCERN score was 2.00 (Q_1_ = 2.00, Q_3_ = 2.00) in all 3 subgroups, with no overall difference (*H* = 0.003, permutation *P* = .965, ε^2^ < 0.001). The median JAMA score was also 2.00 (Q_1_ = 2.00, Q_3_ = 2.00) in all 3 subgroups, and the overall difference was not statistically significant (*H* = 6.701, permutation *P* = .144, ε^2^ = 0.027; Table 4).

**Table 3:** Characteristics, quality, and reliability scores of RD videos by uploader category on TikTok and Bilibili.

Variables	Nonprofessional uploaders (n = 47)	Self-identified professional uploaders (n = 177)	*P* value
General information			
Video length (s), M (Q_1_, Q_3_)	246.00 (111.00, 525.50)	93.00 (63.00, 199.00)	<.001
Likes, M (Q_1_, Q_3_)	56.00 (14.00, 245.50)	185.00 (66.00, 778.00)	<.001
Collections, M (Q_1_, Q_3_)	35.00 (10.00, 146.50)	74.00 (25.00, 276.00)	.009
Comments, M (Q_1_, Q_3_)	25.50 (1.25, 175.75)	18.00 (4.00, 99.00)	.999
Shares, M (Q_1_, Q_3_)	18.00 (4.00, 75.50)	24.00 (7.00, 141.00)	.187
Quality and reliability			
GQS score, M (Q_1_, Q_3_)	2.00 (2.00, 3.00)	2.00 (2.00, 2.00)	.014
mDISCERN score, M (Q_1_, Q_3_)	2.00 (2.00, 2.00)	2.00 (2.00, 2.00)	.317
JAMA score, M (Q_1_, Q_3_)	2.00 (2.00, 2.00)	2.00 (2.00, 2.00)	.002

GQS = Global Quality Scale, JAMA = *Journal of the American Medical Association* benchmark criteria, M = median, mDISCERN = modified DISCERN, Q_1_ = first quartile, Q_3_ = third quartile, RD = retinal detachment.

**Table 4:** Comparison of quality and reliability scores among self-identified professional uploader subgroups.

Variable	Ophthalmologists (n = 169)	TCM ophthalmologists (n = 3)	Other specialists (n = 5)	*H*	Permutation *P*	ε^2^
GQS score, M (Q_1_, Q_3_)	2.00 (2.00, 2.00)	2.00 (2.00, 2.00)	3.00 (2.00, 4.00)	5.756	.040	0.022
mDISCERN score, M (Q_1_, Q_3_)	2.00 (2.00, 2.00)	2.00 (2.00, 2.00)	2.00 (2.00, 2.00)	0.003	.965	<0.001
JAMA score, M (Q_1_, Q_3_)	2.00 (2.00, 2.00)	2.00 (2.00, 2.00)	2.00 (2.00, 2.00)	6.701	.144	0.027

Data are presented as median (Q_1_, Q_3_). Overall comparisons were performed using Monte Carlo permutation-based Kruskal–Wallis tests with 1,000,000 permutations. Pairwise permutation-based Mann–Whitney *U* tests with Holm adjustment were performed for GQS. The adjusted *P* values were .589 for ophthalmologists versus TCM ophthalmologists, .069 for ophthalmologists versus other specialists, and .571 for TCM ophthalmologists versus other specialists.

GQS = Global Quality Scale, *H* = Kruskal–Wallis test statistic, JAMA = *Journal of the American Medical Association* benchmark criteria, M = median, mDISCERN = modified DISCERN, Q_1_ = first quartile, Q_3_ = third quartile, TCM = traditional Chinese medicine, ε^2^ = epsilon-squared effect size.

### 3.4. Video content

As shown in Table 1 and Figure S4, Supplemental Digital Content 7, content analysis of the 224 videos indicated that treatment-related information predominated, accounting for 67.86% of videos. Specifically, 105 TikTok videos and 47 Bilibili videos addressed the treatment of RD. By contrast, topics related to symptoms (48.21%) and prevention in particular (32.59%) comprised a substantially smaller proportion of the material. On TikTok, 47 videos discussed the symptoms of RD, compared with 61 such videos on Bilibili.

### 3.5. Video quality and reliability

Figure S5, Supplemental Digital Content 8, shows that self-identified professional and nonprofessional uploader groups had different distributional patterns for GQS and JAMA despite sharing the same medians. Videos from nonprofessional uploaders showed greater variability in GQS scores, whereas both groups were concentrated in the lower mDISCERN score range. Accordingly, the significant GQS and JAMA results were interpreted as distributional differences rather than as median differences. Overall, both uploader categories exhibited deficiencies in information reliability.

### 3.6. Correlation analysis

**Figure 2.**
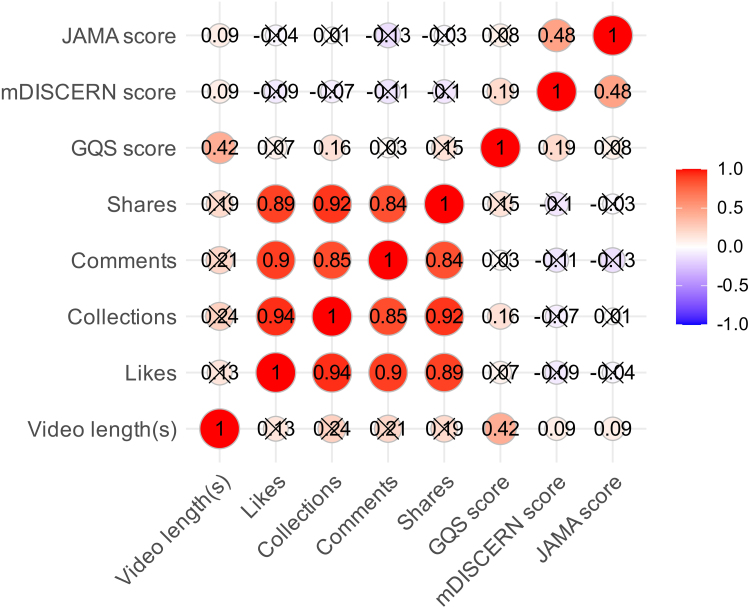
Correlation matrix of Spearman correlation analysis between video metrics and quality scores. GQS = Global Quality Scale, JAMA = *Journal of the American Medical Association* benchmark criteria, mDISCERN = modified DISCERN.

Figure 2 depicts the correlation analysis between video characteristics and video quality. Video duration demonstrated a moderate positive correlation with the GQS score (*r* = 0.42); by contrast, its correlations with the mDISCERN (*r* = 0.09) and JAMA (*r* = 0.09) scores were negligible. Likes, favorites, comments, and shares were highly positively intercorrelated. No significant associations were observed between these engagement metrics and the GQS, mDISCERN, or JAMA score.

## 4. Discussion

This cross-sectional study is the first comprehensive assessment of the quality and reliability of short videos addressing RD on 2 major social media platforms, Bilibili and TikTok. Analysis of 224 videos indicated an overall suboptimal information landscape, with median scores of 2 on the GQS, the mDISCERN instrument, and the JAMA benchmark criteria. We observed significant differences between platforms and among uploader categories in terms of content distribution, engagement metrics, and information quality. These findings are consequential: they underscore a marked discrepancy between the potential of short-video platforms to disseminate critical public health information about sight-threatening emergencies and the current reality of low-quality, inadequately reliable RD content on these platforms. Enhancing the quality of such information is a pivotal step toward enabling patients to seek timely care and improving visual prognosis.

Our analysis of engagement metrics revealed that TikTok videos garnered significantly higher numbers of likes, collections, comments, and shares compared to those on Bilibili, indicating stronger user interaction on the former platform. This finding aligns with studies on other health topics, such as liver cancer and gastroesophageal reflux disease, which also reported higher virality on TikTok.^[16,17]^ However, a pivotal finding of our study was the negligible correlation between these high engagement metrics and the objective quality and reliability scores (GQS, mDISCERN, and JAMA). This dissociation suggests that popularity is not a surrogate for accuracy, a concern similarly raised in assessments of health information on ankle sprains and premature ovarian failure.^[18,19]^ Videos that are emotionally charged, sensationalized, or overly simplistic may accumulate engagement without conveying medically sound information.^[20]^ This underscores a significant risk: patients may be exposed to and influenced by widely disseminated but poor-quality content. Therefore, platforms and health authorities should consider developing and promoting systems that flag or prioritize videos that have been vetted for quality, rather than relying solely on algorithm-driven engagement metrics for content distribution.^[21–23]^

A concerning imbalance was observed in the thematic focus of the videos. Treatment-related information was the most prevalent topic (67.86%), while content covering symptoms (48.21%) and, crucially, prevention (32.59%) was significantly less common. This skew mirrors findings in evaluations of uterine fibroid information on short-video platforms, where treatment topics dominated over symptoms and prevention.^[24]^ For a time-sensitive condition like RD, this content gap is particularly detrimental. The underrepresentation of early warning signs such as sudden floaters, photopsia, and risk factors like high myopia fails to equip the public with the knowledge necessary for early self-recognition and prompt action. As emphasized in previous studies, public awareness of these precursor symptoms is a cornerstone of preventing progression to macula-off detachment and irreversible vision loss.^[6,7,25]^ The scarcity of prevention-focused content represents a missed opportunity for public health intervention.^[4]^ Future health communication initiatives should strategically prioritize creating and amplifying engaging, easy-to-understand content on RD prevention and early symptoms to bridge this critical knowledge gap.

Although both platforms exhibited overall low information quality and shared identical median GQS scores (2.00), subtle differences emerged in their score distributions. Bilibili’s interquartile range for GQS scores (Q_1_ = 2.00, Q_3_ = 3.00) was significantly wider than TikTok’s (Q_1_ = 2.00, Q_3_ = 2.00), indicating greater variability in video quality on the former platform. This finding is consistent with a cross-sectional study on metabolic dysfunction-associated steatotic liver disease information, which also found platform-based variations in content quality.^[26]^ The longer video format typically allowed on Bilibili may provide creators, particularly professionals, with the opportunity to deliver more comprehensive and nuanced explanations. In contrast, TikTok’s short-form, fast-paced nature may incentivize brevity at the expense of depth and accuracy.^[27]^ However, it is vital to note that the reliability scores (mDISCERN and JAMA) did not differ significantly between platforms and were low overall. This indicates that regardless of the platform, fundamental benchmarks of reliable health information, such as clear citation of sources, disclosure of conflicts of interest, and presentation of balanced evidence, are largely unmet. Therefore, merely shifting content creation to longer-format platforms is insufficient. The primary recommendation for both platforms is to integrate and promote the use of standardized, evidence-based reporting tools for health content creators to improve reliability across the board.

Self-identified professional uploaders accounted for 79.02% of the videos in this study, whereas nonprofessional uploaders accounted for 20.98%. Although the median GQS and JAMA scores were identical between the 2 groups, their overall score distributions differed significantly. This finding indicates that the uploader category was associated with differences in the distribution of quality and reliability scores, but it should not be interpreted as evidence of a median difference or uniform superiority of 1 group. Moreover, the generally low scores in both groups suggest that uploader identity alone is insufficient to guarantee high-quality or reliable information. This cautious interpretation is consistent with previous studies showing that uploader identity may be associated with the quality of online medical information, while professional status alone does not ensure adequate reliability.^[28–31]^ An exploratory comparison within the self-identified professional uploader group identified a small overall difference in GQS distributions across professional subgroups. Videos uploaded by other specialists had a higher median GQS than those uploaded by ophthalmologists or ophthalmologists practicing traditional Chinese medicine. However, no pairwise comparison remained statistically significant after adjustment, and the effect size was small. No subgroup differences were detected for mDISCERN or JAMA scores. These findings should be interpreted cautiously because the ophthalmologists practicing traditional Chinese medicine and other specialist subgroups contained only 3 and 5 videos, respectively. The results, therefore, do not support a firm conclusion that 1 professional subgroup consistently produced higher-quality or more reliable content. Platforms should establish credential authentication systems for uploaders and provide science communication training to systematically elevate content standards. When selecting information, viewers should prioritize videos from medical sources and verify citations of authoritative references to mitigate exposure to potential misinformation.^[31]^

Correlation analyses revealed complex interrelationships among video popularity, duration, and quality metrics. Specifically, user engagement indicators demonstrated strong intercorrelations but exhibited no significant associations with any quality assessment scores, reaffirming that popularity serves as an unreliable proxy for information quality.^[32]^ Furthermore, video duration showed a moderate positive correlation with GQS scores, suggesting that longer formats may facilitate the delivery of better-structured and more comprehensive content, a finding consistent with previous research on pancreatic cancer-related videos.^[33]^ However, video length showed negligible correlation with either mDISCERN or JAMA scores. This indicates that merely extending video duration does not inherently enhance core reliability elements such as source attribution or conflict-of-interest disclosure. These critical components must be deliberately and systematically integrated by content creators, independent of video length constraints.

This study has several limitations. First, focusing solely on Bilibili and TikTok limits how broadly our results can apply to global platforms like YouTube or Instagram. Second, although the sample size is comparable to that of similar previous studies, a larger sample would potentially enhance the statistical power for subgroup analyses. Third, despite the video quality assessments being conducted by 2 qualified ophthalmologists using standardized tools, with a third expert adjudicating discrepancies, the process remains inherently subjective. Fourth, the uneven distribution of videos across platforms (Bilibili [n = 82], TikTok [n = 142]) may affect the robustness of cross-platform comparisons. Fifth, although we attempted to corroborate uploader identity using publicly accessible institutional and professional sources, some platform accounts could not be independently linked to a named licensed professional. The uploader classification should therefore be interpreted as being based on publicly available account presentation rather than formal verification of professional licensure, and residual misclassification cannot be entirely excluded. Sixth, the professional subgroup analysis was exploratory, and the small numbers of videos from ophthalmologists practicing traditional Chinese medicine and other specialists limited statistical power. Future research could address these limitations by incorporating more platforms, expanding sample sizes, and employing more objective assessment technologies.

## 5. Conclusion

This study illuminates the current state of RD information on short-video platforms in China, characterizing it as a field with significant room for improvement. The overall low quality and reliability, the skewed content distribution, and the disconnect between engagement and accuracy collectively pose a risk to patient education. We urge collaborative efforts among healthcare professionals, platform regulators, and public health bodies to establish quality control standards, promote evidence-based content creation, and educate the public on critically evaluating online health information. Such initiatives are essential to harness the power of social media for improving early detection of vision-threatening conditions like RD.

## Acknowledgments

The authors thank video creators for their public health contributions.

## Author contributions

**Conceptualization:** Shengjin Tu, Jingtao Huang, Jinyang Peng.

**Formal analysis:** Shengjin Tu, Kaiyin Tan, Jinyang Peng.

**Methodology:** Shengjin Tu, Kaiyin Tan, Jinyang Peng.

**Project administration:** Shengjin Tu.

**Supervision:** Shengjin Tu.

**Visualization:** Shengjin Tu.

**Writing – review & editing:** Shengjin Tu.
















