# Supplementary material for: Evaluating the reliability and quality of retinal detachment educational content on TikTok and Bilibili: A cross-sectional content analysis
Source: Medicine (Baltimore). 2026 Aug 14;105(33):e50274. doi: 10.1097/MD.0000000000050274 (PMC13480779; doi:10.1097/MD.0000000000050274)
Supplement: Supplementary file 2 [file medi-105-e50274-s003.docx]

**Supplementary Table S2.** The Modified DISCERN (mDISCERN) quality criteria.

**Reliability Score**

1. Is the video clear, concise, and understandable?

2. Are valid sources cited?

3. Is the content presented balanced and unbiased?

4. Are additional sources of content listed for patient reference?

5. Are areas of uncertainty mentioned?

* (1 point for answer ‘yes’, 0 point for answer ‘no’)
