# Supplementary material for: Evaluating the reliability and quality of retinal detachment educational content on TikTok and Bilibili: A cross-sectional content analysis
Source: Medicine (Baltimore). 2026 Aug 14;105(33):e50274. doi: 10.1097/MD.0000000000050274 (PMC13480779; doi:10.1097/MD.0000000000050274)
Supplement: Supplementary file 3 [file medi-105-e50274-s004.docx]

**Supplementary Table S3**. The Journal of the American Medical Association (JAMA) benchmark criteria.

| Score* | Score component | |
| --- | --- | --- |
| 1 score | Authorship | Author and contributor credentials and their affiliations should be provided. |
| 1 score | Attribution | Clearly lists all copyright information and states references and sources for content. |
| 1 score | Currency | Initial date of posted content and subsequent updates to content should be provided. |
| 1 score | Disclosure | Conflicts of interest, funding, sponsorship, advertising, support, and video ownership  should be fully disclosed. |

*The criteria of each aspect were scored separately, with 1 point for each criterion, with a total score of 4 points.
